#  Thrombelastography and tromboelastometry in assessing coagulopathy in trauma

**DOI:** 10.1186/1757-7241-17-45

**Published:** 2009-09-23

**Authors:** Pär I Johansson, Trine Stissing, Louise Bochsen, Sisse R Ostrowski

**Affiliations:** 1 Section for Transfusion Medicine, Regional Blood Bank, Rigshospitalet, University of Copenhagen, Denmark

## Abstract

Death due to trauma is the leading cause of lost life years worldwide, with haemorrhage being responsible for 30-40% of trauma mortality and accounting for almost 50% of the deaths the initial 24 h. On admission, 25-35% of trauma patients present with coagulopathy, which is associated with a several-fold increase in morbidity and mortality. The recent introduction of haemostatic control resuscitation along with emerging understanding of acute post-traumatic coagulability, are important means to improve therapy and outcome in exsanguinating trauma patients. This change in therapy has emphasized the urgent need for adequate haemostatic assays to monitor traumatic coagulopathy and guide therapy. Based on the cell-based model of haemostasis, there is emerging consensus that plasma-based routine coagulation tests (RCoT), like prothrombin time (PT) and activated partial thromboplastin time (APTT), are inappropriate for monitoring coagulopathy and guide therapy in trauma. The necessity to analyze whole blood to accurately identify relevant coagulopathies, has led to a revival of the interest in viscoelastic haemostatic assays (VHA) such as Thromboelastography (TEG^®^) and Rotation Thromboelastometry (ROTEM^®^). Clinical studies including about 5000 surgical and/or trauma patients have reported on the benefit of using the VHA as compared to plasma-based assays, to identify coagulopathy and guide therapy.

This article reviews the basic principles of VHA, the correlation between the VHA whole blood clot formation in accordance with the cell-based model of haemostasis, the current use of VHA-guided therapy in trauma and massive transfusion (haemostatic control resuscitation), limitations of VHA and future perspectives of this assay in trauma.

## Introduction

On admission, 25-35% of trauma patients present with coagulopathy, which is associated with a several-fold increase in morbidity and mortality [1,2]. Although the management of traumatic coagulopathy differs worldwide [3,4], the recent introduction of haemostatic control resuscitation [5-7] and the emerging understanding of acute post-traumatic coagulopathy [1,2,8,9], emphasize the urgent need for adequate haemostatic assays to guide therapy. Classically, coagulopathy is often monitored by plasma-based routine coagulation tests (RCoT) such as activated partial thromboplastin time (APTT) and prothrombin time (PT). These assays were developed half a century ago to monitor haemophilia and anticoagulation therapy, but have unfortunately never been validated for the prediction of haemorrhage in a clinical setting [10,11]. It should be noted that although abnormal PT and APTT are highly correlated with mortality in trauma patients, the cause of death in these patients is not identified as excessive bleeding [12-14]. This lack of correlation with clinically relevant coagulopathies can be explained by the fact that plasma-based assays reflect only the small amount of thrombin formed during initiation of coagulation [15,16]. Consequently, recent reviews have concluded that the plasma-based assays are inappropriate for monitoring coagulopathy or guide transfusion therapy, calling for new tests to monitor these complex patients [17,18].

In 1994, the classical clotting cascade of haemostasis [19,20] was challenged by the introduction of a cell-based model of haemostasis emphasizing the importance of tissue factor (TF) as the initiator of coagulation and the pivotal role of platelets for intact haemostasis [21]. The poor correlation between RCoT and clinical bleeding in e.g. trauma and surgery [12-15,22-25] is, hence, explained by this new understanding of haemostasis.

The necessity to analyze whole blood to accurately identify relevant coagulopathies, has led to a revival of the interest in viscoelastic point-of-care haemostatic assays (VHA) such as Thromboelastography (TEG^®^) and Rotation Thromboelastometry (ROTEM^®^).

The objective of this article is to review the basic principles of VHA, the correlation between the result of VHA and clot formation in accordance with the cell-based model of haemostasis, the current use of VHA-guided therapy in trauma and massive transfusion (haemostatic control resuscitation), limitations of VHA and future perspectives of application of this assay.

### Basic principles of VHA

**Figure 1:**
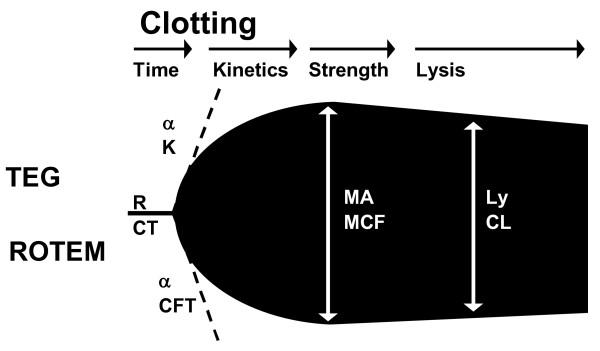
**Schematic TEG (upper part)/ROTEM (lower part) trace indicating the commonly reported variables reaction time (R)/clotting time (CT), clot formation time (K, CFT), alpha angle (α), maximum amplitude (MA)/maximum clot firmness (MCF) and lysis (Ly)/clot lysis (CL)**.

**Figure 2:**
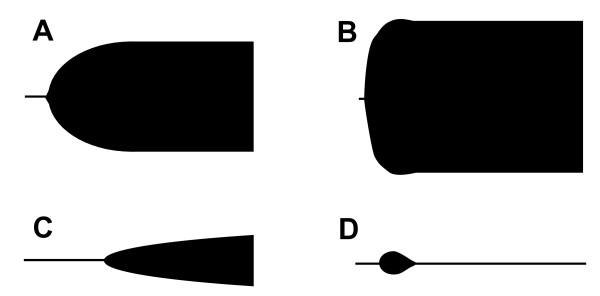
**Schematic presentation of various VHA tracings**: A) Normal, B) Hypercoagulability, C) Hypocoagulability (thrombocytopenia/pathy) and D) Primary hyperfibrinolysis.

Thrombelastography was first described in 1948 by H. Hartert [26], as a method to assess the viscoelastic properties of coagulation in whole blood under low shear conditions [27-31]. The VHA gives a graphic presentation of clot formation and subsequent lysis. Blood is incubated at 37°C in a heated cup. Within the cup is suspended a pin connected to a detector system (a torsion wire in TEG and an optical detector in ROTEM). The cup and pin are oscillated relative to each other through an angle of 4° 45'. The movement is initiated from either the cup (TEG) or the pin (ROTEM). As fibrin forms between the cup and pin, the transmitted rotation from the cup to pin (TEG) or the impedance of the rotation of the pin (ROTEM) are detected at the pin and a trace generated (Figure 1). The trace is divided into parts that each reflects different stages of the haemostatic process (clotting time, kinetics, strength and lysis, Figure 1) with slightly different nomenclature for TEG and ROTEM (Table 1). Examples of traces generated from normal as compared to different pathological states are shown in Figure 2.

**Table 1:** Nomenclature of TEG and ROTEM

Parameter	TEG^®^	ROTEM^®^
Clot time		
Period to 2 mm amplitude	R (reaction time)	CT (clotting time)
Clot kinetics		
Period from 2-20 mm amplitude	K (kinetics)	CFT (clot formation time)
α-angle	α (slope between R and K)	α (slope of tangent at 2 mm amplitude)
Clot strength		
Maximum strength	MA (maximum amplitude)	MCF (maximum clot firmness)
Clot elasticity	G	MCE (maximum clot elasticity)
Clot lysis		
Lysis (at fixed time)	Ly30, Ly60 (amplitude reduction 30/60 min after MA)	CL30, CL60 (amplitude reduction 30/60 min after MCF)

VHA can either be performed bedside using native non-anticoagulated blood if the sample is analyzed within 5 min or it can be performed in a laboratory setting, where citrated blood samples are employed [32]. The technical stability of the VHA analysis is demonstrated by day-to-day variation (CV%) of 5-15% for the different parameters [32,33].

Compared to RCoT, VHA has several advantages. First, the evaluation of the coagulation system in whole blood allows assessment of the combined influence of circulating plasmatic and cellular (platelets, RBC, leukocytes) elements on clot formation, including platelet function. Second, the end-point is clinically relevant, i.e. clotting in whole blood (fibrin formation, clot retraction and fibrinolysis, Figure 2). Third, the results are available within a short time frame making them relevant to clinical decision-making.

## VHA and the cell-based model of haemostasis

According to the cell-based model, haemostasis is described in three phases [34-36]: *Initiation*, *amplification *and *propagation*. During *initiation*, circulating activated coagulation factor (F) VII (FVIIa) forms a complex with exposed TF on injured endothelium, which in the amplification stage generates a small amount of thrombin that mainly activates the platelets. In the *propagation *phase the coagulation factors assemble on the activated platelets generating large amounts of thrombin ("thrombin burst"). The rate and peak of thrombin generation influences the clot structure and stability [37], by activating FXIII to FXIIIa, which cross links fibrinogen and further stabilizes the clot [38]. Furthermore, thrombin activates TAFI to TAFIa which prevents lysis of the fibrin clot [39].

The three different phases of cell-based haemostasis resulting in clot formation are reflected by the VHA. The structural changes in the clot along the VHA trace was recently investigated by scanning electron microscopy demonstrating that the R (TEG)/CT (ROTEM) corresponds to the initiation phase whereas K (TEG)/CFT (ROTEM) reflects the amplification phase [40]. Our group and others have demonstrated that the thrombin burst is reflected by the α-angle (TEG/ROTEM), and determines the clot strength and stability [41,42]. The ability of VHA to reflect thrombin generation has profound clinical utility because coagulation factor deficiencies secondary to e.g. massive bleeding, dilution, consumption and thrombocytopenia/pathy result in impaired thrombin generation and impaired clot strength (MA (TEG), MCF (ROTEM)) [30,43]. The whole blood based VHA, therefore, reveals the contribution of all circulating plasmatic and cellular components, in their actual concentrations, to clot formation [44]. Importantly, enhanced fibrinolysis contributes significantly to bleeding in trauma patients as well as patients undergoing cardiac and liver surgery and patients with obstetric complications, and this condition is readily identified by VHA (Ly (TEG), CL (ROTEM)) [45]. In addition, VHA *in vitro *studies have evaluated the effects of hypothermia [46], acidosis [47], different crystalloids and colloids [48], pro-haemostatic [49] and anti-fibrinolytic drugs [50], with results being highly relevant for the clinical setting.

## VHA in the surgical setting

In the last 25 years, more than 20 clinical studies reporting on the benefit of using VHA when compared to RCoT to identify coagulopathy and guide transfusion therapy have been published (Table 2). The studies include three randomized clinical trials and involve more than 4,500 patients undergoing major surgery. The majority of studies have been performed in patients undergoing liver or cardiac surgery [51-57], all reporting of the benefit of using VHA when compared to RCoT, evidenced by reductions in transfusion requirements and need for re-exploration and improved ability to predict the need for blood transfusion in patients with VHA-guided therapy. Importantly, no study have to date reported a benefit of employing plasma-based RCoT to predict bleeding or guide transfusion therapy, when compared to VHA, supporting the scientific rationale of whole blood viscoelastic assays in this setting.

**Table 2:** Studies evaluating the effect of TEG vs. routine coagulation tests (RCoT) on haemostasis in surgical patients

Author	Patients	No.	Study type	Major conclusions
Kang (1985)	Liver surgery	66	RC	VHA based therapy reduced blood and fluid infusion volume by 33% vs. RCoT therapy
McNicol (1994)	Liver surgery	75	RC	VHA enabled specific and selective use of FFP, PLT and cryoprecipitate
Kang (1995)	Liver surgery	80	RC	VHA identified clinically relevant fibrinolysis and enabled specific pharmacological therapy
Harding (1997)	Liver surgery	55	RC	VHA-heparinase enabled identification of coagulopathy present under the heparinisation
Chau (1998)	Liver surgery	20	PO	VHA predicted re-bleeding in cirrhotic patients with variceal bleeding, whereas RCoT did not
Tuman (1987)	Cardiac surgery	87	RC	VHA allowed rapid intraoperative diagnosis of coagulopathy during CPB
Spiess (1987)	Cardiac surgery	38	RC	VHA was a better predictor (87% accuracy) of postoperative haemorrhage and need for reoperation than RCoT (30-51% acuracy)
Tuman (1989)	Cardiac surgery	42	RC	VHA, but not RCoT, predicted postoperative bleeding in patients post-CPB
Essell (1993)	Cardiac surgery	36	PO	VHA had higher specificity in predicting patients likely to benefit from FFP and PLT therapy than RCoT
Tuman (1994)	Cardiac surgery	51	RC	VHA-heparinase revealed post-CPB coagulopathy
Spiess (1995)	Cardiac surgery	1,079	PI vs. RC	VHA guided transfusion therapy significantly reduced overall incidence of transfusion and total transfusions in the OR as compared to RCoT
Shih (1997)	Cardiac surgery	43	RC	VHA demonstrated higher sensitivity and specificity than RCoT for detecting post-CPB bleeding
Cherng (1998)	Cardiac surgery	74	RC	Re-do patients demonstrated reduced pre-operative α-angle and MA/MCF was significantly reduced compared to patients not needing re-exploration
Shore-Lesserson (1999)	Cardiac surgery	105	RCS	VHA treated patients received fewer postoperative FFP and PLT transfusions than patients treated based on PCoT
Royston (2001)	Cardiac surgery	90	IS	VHA guided transfusion therapy reduced the need for FFP and PLT threefold vs. RCoT
Manikappa (2001)	Cardiac surgery	150	RCS	VHA had higher accuracy than RCoT to predict patients developing excessive postoperative bleeding and significantly reduced the need for RBC, FFP and PLT transfusions
Welsby (2006)	Cardiac surgery	30	PO	VHA MA/MCF showed better correlation with postoperative bleeding than RCoT
Anderson (2006)*	Cardiac surgery	990	PI vs. RC	VHA guided therapy reduced the need for RBC, FFP and PLT as compared to RCoT directed therapy
Westbrook (2008)	Cardiac surgery	69	RC	VHA-based management reduced total product usage by 58.8% in the study group vs. RCoT group
Reinhöfer (2008)*	Cardiac surgery	150	RC	Clot strength, but not RCoT, had the highest predictive value for excess postoperative blood loss
Johansson (2009)	Massive transfusion	832	PI vs. RC	VHA guided therapy reduced mortality from 31% to 20% in massively bleeding patients

*ROTEM, RCoT = routine coagulation tests, PO = Prospective observational study, RC = Retrospective cohort study, RCS = Randomised clinical study, PI vs. RC = Prospective interventional study vs. retrospective controls, IS = Interventional study

### Massive transfusion

Our group reported the effect on mortality of guiding transfusion therapy in massively bleeding patients (n = 832, 21% trauma patients) by VHA as compared to RCoT. Patients treated according to the VHA results received more FFP and more platelets and had significantly lower 30-day mortality as compared to controls (20% vs. 32%) [58].

It is intriguing that the increased amount of plasma and platelets administered based on the VHA results are associated with improved survival, in alignment with retrospective findings from the trauma setting [59] as well as the implementation of blind transfusion protocols [60].

## VHA in trauma

A conserved physiologic haemostatic response, characterized by immediate activation of coagulation and fibrinolysis followed by subsequent fibrinolytic shutdown and later reactivation is often observed in trauma patients [61]. Post-traumatic coagulopathy is classically described as dysfunction and/or consumption of coagulation factors and platelets due to dilution, hypothermia and acidosis i.e., "the bloody viscious cycle" and the ability of VHA to identify these conditions has been extensively reported [22,23,58,62-70]. At present, 10 studies including more than 700 patients have evaluated VHA in trauma patients (Table 3).

**Table 3:** Studies evaluating VHA in trauma patients

Author	No.	ISS	Study type	Major conclusions	Ref.
Kaufman (1997)	69	13/29	RS	Moderately injured patients (ISS 13) were hypercoagulable whereas severely injured (ISS 29) patients were hypocoagulable according to VHA	[51]
Schreiber (2005)	65	23	RS	62% of the patients where hypercoagulable 1^st ^day of trauma according to VHA which is more sensitive to identify this state than RCoT.	[52]
Rugeri (2007)	90	22	PO	VHA rapidly detects systemic changes of *in vivo *coagulation in trauma patients, and it might be a helpful device in guiding transfusion.	[76]
Plotkin (2008)	44	21	RS	VHA is a more accurate indicator of transfusion requirements than PT, APTT and INR	[77]
Levrat (2008)	87	20/75	PO	VHA provides rapid and accurate detection of hyperfibrinolysis in severely injured trauma patients	[78]
Schöchl (2009)	33	47	PO	VHA based diagnosis of hyperfibrinolysis predicted outcome in severely injured trauma patients	[79]
Carroll (2009)	161	20	PO	Abnormal VHA parameters correlated with fatality. Coagulopathy as evaluated by VHA was present already on the scene of accident.	[80]
Jaeger (2009)	20	??	RS	RapidTEG provides earlier detection of coagulopathy than standard VHA and RCoT	[81]
Park (2009)	78	20	PO	VHA detected hypercoagulability and this was not seen with RCoT in trauma patients	[82]
Kashuk (2009)	44	29	RS	RapidTEG may effectively guide transfusion therapy in trauma patients	[83]

RCoT = routine coagulation tests, RS = Retrospective study, PO = Prospective observational study

Kaufmann *et al*. found that in 69 patients with blunt trauma, 65% displayed hypercoagulability upon arrival at the emergency department (ED) whereas only 10% were hypocoagulable. Interestingly, a hypocoagulable TEG was associated with increased ISS and only ISS and VHA, not RCoT, was predictive for early transfusion [62]. Schreiber and colleagues [63] also found that hypercoagulability, as evaluated by VHA, was frequent (62%) in trauma patients (n = 65) upon arrival at the ED, and that this correlated with increased thrombin-antithrombin (TAT) complex generation. APTT, PT and platelet count where within normal limits and could, hence, not identify a hypercoagulable state. Rugeri and colleagues [64] investigated 88 trauma patients and compared their VHA results with that of healthy subjects. They found that trauma patients demonstrated evidence of hypocoagulability, and that this was restricted to those trauma patients also being coagulopathic with RCoT.

Recently, Carroll and colleagues [65] addressed the acute post-traumatic coagulopathy, reported by Brohi *et al*., [2,8,9] by VHA analyses of samples obtained at the scene of accident and upon arrival in the ED in 161 trauma patients. Interestingly, they found that that the clot forming parameters demonstrated hypocoagulability and correlated with fatality, whereas none of the RCoT demonstrated such a correlation. This indicates that VHA is more sensitive in reflecting clinically relevant coagulopathies than RCoT. This has important implications, since the VHA result is available within a short time frame as interventions aiming at normalising the VHA profile and hence the coagulopathy, can be instituted early during resuscitation. The VHA results of acute post-traumatic coagulopathy presented by Carroll *et al*. do not, however, corroborate the frequency of hyperfibrinolysis reported by Brohi *et al*. [8]. Only three patients (2%) demonstrated evidence of increased fibrinolysis compared to the hyperfibrinolysis described in the cohort of Brohi, using D-dimer as a marker of fibrinolysis. Levrat and colleagues [66] reported in a cohort of 89 trauma patients that 5 (6%) showed evidence of increased fibrinolysis and that this correlated with euglobuline lysis time. In both the study of Carroll *et al*. and Levrat *et al*., hyperfibrinolysis was identified in the most severely injured patients and was associated with increased mortality rate confirming that although rare, this is a very serious condition. A unique feature of VHA is its ability to identify patients with increased fibrinolysis. This enables initiation of specific anti-fibrinolytic therapy, which is associated with decreased blood loss and/or transfusion requirements in non-trauma settings [71]. The role of this therapy in trauma patients [72] is currently under clinical evaluation http://www.crash2.lshtm.ac.uk/.

In a retrospective review of 44 combat patients with penetrating trauma Plotkin *et al*. [23] reported that VHA was a more accurate indicator of blood product requirements than PT, APTT, and INR. They suggested that VHA aided by platelet count and haematocrit should guide blood transfusion requirements. This is in alignment with Martini and colleagues [22] who demonstrated that VHA was superior than PT, APTT, and Activated Clotting Time in detecting clinically relevant clotting abnormalities after hypothermia, haemorrhagic shock and resuscitation in pigs.

Recently Jaeger and colleagues [67] reported of a modification of the VHA (TEG) where the activator kaolin was substituted with TF (RapidTEG). In patients sustaining major blunt trauma they investigated the time from ED arrival to the results of standard TEG, RapidTEG and RCoT were available. RapidTEG was available significantly faster (19.2 min vs. 29.9 min for kaolin TEG and 34.1 min for RCoT). On average the time until the results were available was reduced by approximately 50% for RapidTEG as compared to standard TEG, which may be of clinical relevance.

## VHA limitations

Important limitations of the VHA exist and should be taken into consideration when interpreting the results of the analysis. Firstly, though it is possible to adjust the temperature at which the blood sample is analysed, VHA is routinely performed at 37°C and therefore the effect of hypothermia will not be recognised [73,74]. Secondly, the coagulation activators employed results in thrombin formation, which masks the possible inhibition that antithrombotic agents such as aspirin, NSAID, clopidogrel and eptifibatide may have on the platelets ability to aggregate [75]. Consequently, a normal VHA profile does not rule out clinically significant platelet inhibition. Thirdly, the endothelial contribution to haemostasis is not displayed in VHA and therefore, conditions affecting the endothelium such as von Willebrand disease (vWD, quantitative or qualitative defects in vWF and, hence inability of the platelets to adhere to the endothelium), cannot be investigated. If these causes of abnormal bleeding can be excluded, then a normal VHA trace along with clinically significant bleeding necessitating blood transfusion is suspect of a surgical cause. Thus, our group found 97% predictability by VHA in identifying a surgical cause of bleeding in postoperative non-cardiac patients with ongoing transfusion requirements [68].

## VHA future perspectives in trauma

Recently, the concept of acute traumatic coagulopathy (ATC) was introduced by Brohi et al. [2,8,9,13] based on the observations that coagulopathy, as evaluated by increased PT, APTT and D-dimer levels, was present in trauma patients already upon arrival to the hospital. ATC was independent of traditional causes of coagulopathy but occurred only in patients with evident hypoperfusion. When evaluating trauma patients upon arrival at ED with VHA characteristic profiles are found that are related to ISS and mortality. In patients with minor trauma/tissue injury a normal VHA trace is seen (Figure 2A) whereas in patients with moderate trauma (ISS between 10-20) hypercoagulability is seen (Figure 2B). In patients with severe injury (ISS 20-35), an increased frequency of hypocoagulability is seen (Figure 2C) whereas patients with massive tissue injury (ISS above 30) hyperfibrinolysis is seen (Figure 2D). The different VHA traces indicate that different treatment strategies may be appropriate and this warrants further investigation.

## Conclusion

Death due to trauma is the leading cause of lost life years worldwide, with haemorrhage being responsible for 30-40% of trauma mortality and accounting for almost 50% of the deaths the initial 24 h [5]. There is emerging consensus that plasma-based assays are inappropriate for monitoring coagulopathy and guide transfusion therapy in trauma [17,18], and the cell-based model of haemostasis [21,34-36] provides a reliable explanation for this notion. Clinical studies including more than 5000 surgical and/or trauma patients have reported on the benefit of using VHA when compared to RCoT to identify coagulopathy and guide transfusion therapy. However, at present no VHA guided transfusion therapy has been prospectively and independently validated in trauma patients, which is highly warranted.

## Competing interests

PJ has received unrestricted research grants from Haemoscope Corp. Niles IL, USA. The other authors declare that they have no competing interests'.

## Authors' contributions

PJ, SO conducted the MEDLINE search for relevant publications related to VHA. PJ, SO, LB, TS conducted review of the searched publications and jointly decided which to be included in the review. PJ, SO wrote the first draft of the manuscript. SO designed the figures for the manuscript. PJO SO, LB, TS developed the tables. All authors read and approved the final manuscript.
